# Subcutaneous phaeohyphomycosis caused by *Aureobasidium Pullulans* in an immunocompetent carpenter

**DOI:** 10.1016/j.mmcr.2022.02.003

**Published:** 2022-02-16

**Authors:** Sofia Alami, Imane Sekkal, Sarra Aoufi, Mohammed Lyagoubi, Laila Benzekri, Karima Senouci

**Affiliations:** a Dermatology Department, Ibn Sina University Hospital Center, Rabat, 10000, Morocco; b Central Laboratory of Parasitology and Mycology, Ibn Sina University Hospital Center, Rabat, 10000, Morocco

**Keywords:** Phaeohyphomycosis, Aureobasidium pullulans, Dematiaceous fungi, Subcutaneous mycosis

## Abstract

Phaeohyphomycosis refers to uncommon infections due to a large group of heterogeneous organisms called “dematiaceous fungi”. Here, we report a rare case of subcutaneous phaeohyphomycosis in an immunocompetent carpenter, presenting as multiple verrucous and confluent papulo-nodules of the right leg, and likely due to traumatic inoculation. The pathogenic fungal species was identified as *Aureobasidium pullulans*, according to macroscopic and microscopic morphological characteristics of the colonies. Surgical excision of the entire lesion and adjunctive antifungal therapy was curative.

## 1 Introduction

*Aureobasidium pullulans* is a dematiaceous fungus that is found in plant debris, oil, wood, textiles, and indoor air. Various infection sites have been described as reported cases, including peritonitis (among patients on peritoneal dialysis), cutaneous infection, pneumonia, meningitis, corneal and scleral infection, abscesses in the spleen and jaw, as well as systemic infection. However, the pathogenicity of *A. pullulans* remains limited and infections rare, which affect mainly immune-compromised hosts [1]. We report a case of subcutaneous phaeohyphomycosis in an immunocompetent patient.

### 1.1 Case presentation

**Fig. 1:**
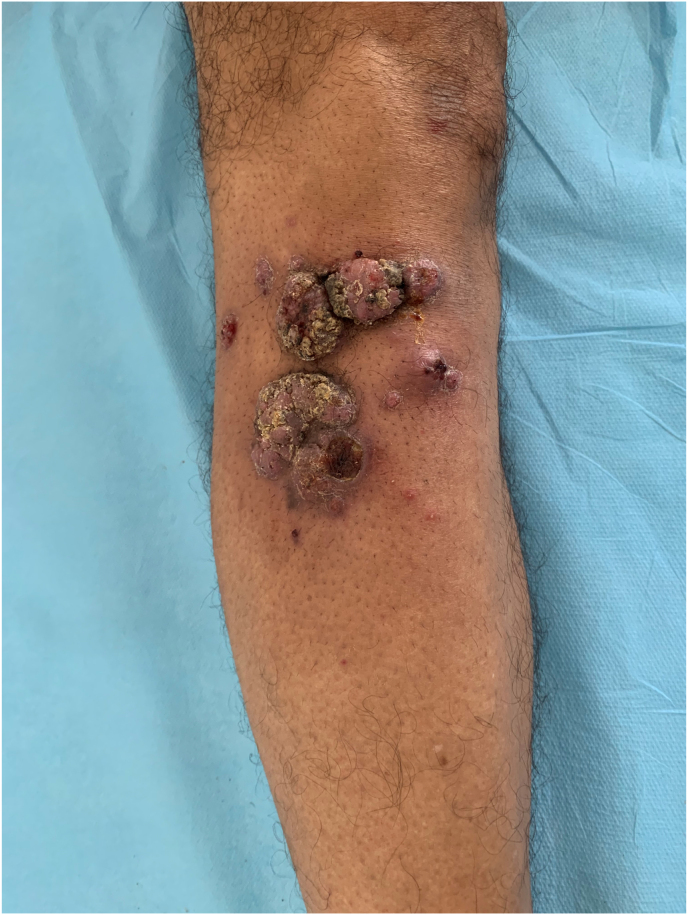
Multiple verrucous and confluent violaceous papulo-nodules surrounded by yellow keratin on the front side of the right leg.

**Fig. 2:**
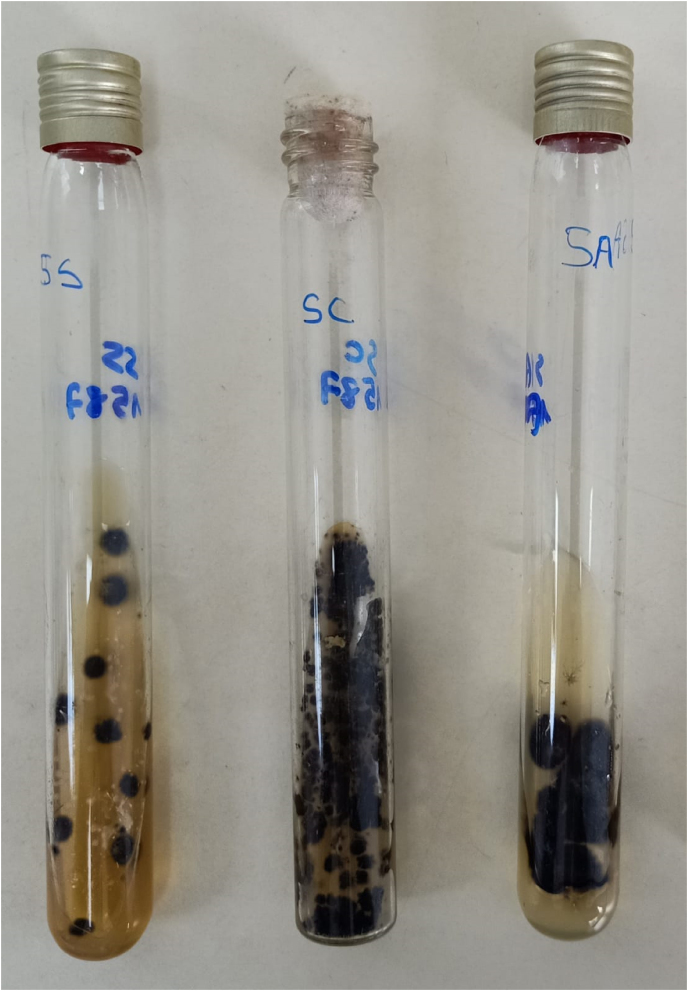
Growth of Aureobasidium Pullulans in culture showing characteristic black colored colonies (From left to right: Sabouraud's Dextrose Agar (SS), Sabouraud's Chloramphenicol Agar (SC) and Sabouraud’s Chloramphenical Actidion Agar (SCA)).

**Fig. 3:**
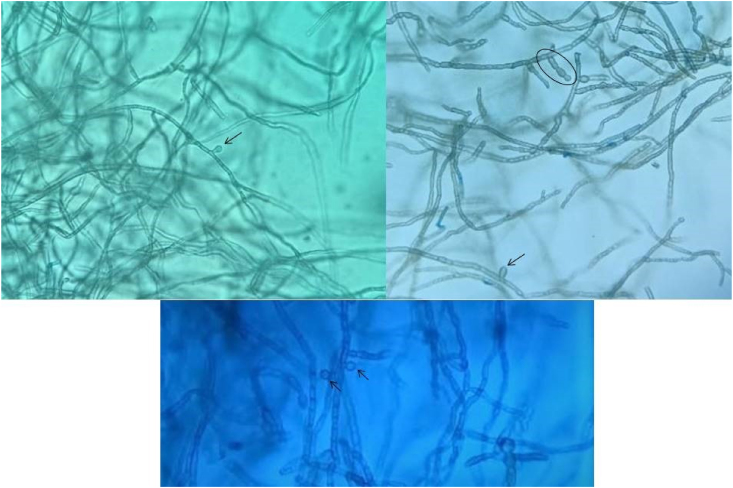
Aureobasidium pullulans seen under microscopy using lactophenol cotton blue stain (40x magnification) showing thick septate hyphae with conidia produced directly from hyphae (black arrows), and chlamydospores (black circle).

A previously healthy 66-year-old carpenter presented with a 16-month history of slowly growing nodules over his right leg. He indicated that the lesions started following a skin trauma with a piece of wood. At the day of presentation (day 0), physical examination revealed three principals violaceous and verrucous nodules surrounded by several littles keratotic papulo-nodules (Fig. 1). The lesions were neither painful nor itchy. Results of full blood count and routine blood chemistry were normal, and serology for human immunodeficiency virus, hepatitis B virus and hepatitis C virus was negative. Histopathology analysis of an incisional biopsy (day +15) showed a noncaseating granulomatous dermatitis. Testing for *Mycobacterium tuberculosis*, including Ziehl Neelsen smear and Lowenstein-Jensen culture were negative. Direct examination in potassium hydroxide preparation of a biopsy sample revealed the presence of fungal hyphae. The material was seeded on culture in Sabouraud's Dextrose Agar (SC), Sabouraud's Chloramphenicol Agar (SC) and Sabouraud’s Chloramphenical Actidion Agar (SCA), and incubated at 27 °C and 37 °C. After 6 days, growth in all media showed moist and smooth black colonies with a pale reverse (Fig. 2). Microscopic examination of a part of the colony revealed hyaline to brown, thin-walled and arthrosporated hyphae, with some pear-shaped conidia produced directly from hyphae (Fig. 3). It was identified as Aureobasidium pullulans according to morphological characteristics. There were no deep tissue involvement in magnetic resonance imaging of the leg and tomography of the thorax, abdomen, and pelvis ruled out systemic compromise. He was diagnosed with subcutaneous localized phaeohyphomycosis. Surgical shaving was performed, and he was treated with itraconazole 200 mg/d for 2 months (day +56). Complete healing was achieved at the end of the treatment, which only left a dyschromic scar (day + 87).

## 2 Discussion

Phaeohyphomycosis refers to infections due to a large group of heterogenous organisms called “dematiaceous”, or “darkly pigmented” fungi. The most common isolate is Exophiala jeanselmei, followed by Wangiella derrnalil [2], while A. pullulans is very unusual. Most individuals are exposed to dematiaceous fungi through inhalation, as they are ubiquitous in the environment, although the development of infection is extremely rare [3], usually reported in immunocompromised hosts.

Aside from its worldwide distribution, A. pullulans is generally considered as a contaminant of biological samples [4]. However, in the present case, its isolation in three different skin biopsies, and the negativity of mycobacterial cultures, leads us to consider this agent as a pathogen. Given the natural habitat of this fungus, we can consider that our patient's profession (that constantly exposes him to wood) as well as history of previous skin trauma, are both triggering factors for cutaneous phaeohyphomycosis. A reviewed of the Indian literature on subcutaneous pheohyphomycosis showed that, as in the present case, 66% of patients had lesions on the limbs, supporting the hypothesis of traumatic skin wound [5]. It was also noted that the penetrating injury mechanism occurs in most cases in immunocompetent people [6]. In this case, the fungus is inoculated deep into the dermis, thus causing a real inflammatory reaction. In the days or months following inoculation, a macular lesion appears, followed by a papule, which develops into one or more violaceous dermal nodules. The phaeohyphomycotic lesion then becomes scaly and crusty [7].

In its cutaneous form, paeohyphomycosis can be divided into 3 distinct groups: a) superficial: including black piedra and tinea nigra; b) cutaneous: phaeomycotic dermatomycosis and onychomycosis, due to colonization and proliferation of the fungi in keratinized tissues, and c) subcutaneous: as in the present case, with an implication of the dermis or even the hypodermis [2]. Some overlap exists between the cutaneous and subcutaneous forms. The most common lesion is a subcutaneous cyst on the distal parts of the extremities [8], which is also the main location to external injuries. Others clinical presentations, that are non-specific, have been reported, including papulonodules, verrucous, hyperkeratotic or ulcerated plaques, abscesses, pyogranuloma, non-healing ulcers or sinuses [9].

In our patient described here, the diagnosis of phaeohyphomycosis was based on the colonial morphology and its microscopic aspect, with affirmation of its pathogenic nature (tissue reaction of the host) as opposed to "contamination" of a biological fluid or superficial "colonization" of a soiled tissue. Colonies of A. Pullulans are fast growing, maturing into a cottony, fluffy colony which may darken with age. In microscopy, hyphae are septate, at first appearing hyaline, then with age becoming dark and thick walled. A. Pullulans can be recognized by conidia, that are are round, oval, to pear-shaped and produced directly from the hyphae, and by the presence of lobed chains of thick-walled chlamydospores [10]. However, the identification of the species can be difficult and the contribution of molecular diagnosis is then very helpful to the diagnosis [7].

The prognosis of A. pullulans infection depends on the extent of infections and host conditions. In the immunocompetent host, localized cutaneous phaeohyphomycosis is usually treated by complete surgical excision. Oral systemic therapy with an azole antifungal agent in conjunction with surgery is frequently employed and has been used successfully. The agents that have most frequently been used include amphotericin B, itraconazole, and terbinafine. Antifungal treatment durations are individualized but often last between 4 and 8 weeks, or longer [11].

In conclusion, we present a unique case of chronic verrucous nodules of the leg, found to be a subcutaneous phaeohyphomycosis due to A. Pullulans. The diagnosis was based on the contribution of parasitological samples coupled with the presence of granulomatous reaction in histopathologic examination, excluding the possibility of a simple colonization. In immunocompetent patient, surgical excision of the entire lesion, whether or not combined with adjunctive antifungal therapy, is usually curative.

## Conflict of interest

There are none.
